# Quantitative resistance to *Rhizoctonia solani* AG-4 develops independently in leaves and roots of *Brachypodium distachyon*

**DOI:** 10.3389/fpls.2026.1875734

**Published:** 2026-07-03

**Authors:** Rozi Fernanda, Niranjan Mahadevan, Natsuka Kohno, Reiko Nagao, Megumi Watanabe, Nanami Sakata, Hidenori Matsui, Kazuhiro Toyoda, Yuki Ichinose, Yusuke Kouzai, Keiichi Mochida, Yoshiteru Noutoshi

**Affiliations:** 1 Graduate School of Environmental, Life, Natural Science and Technology, Okayama University, Okayama, Japan; 2 Tea Research Institute of Sri Lanka, Talawakelle, Sri Lanka; 3 School of Agriculture, Okayama University, Okayama, Japan; 4 Crop Stress Management Group, Division of Plant Molecular Regulation Research, Institute of Agrobiological Sciences, National Agriculture and Food Research Organization (NARO), Tsukuba, Japan; 5 RIKEN Center for Sustainable Resource Science, Yokohama, Japan; 6 Kihara Institute for Biological Research, Yokohama City University, Yokohama, Japan; 7 School of Information and Data Sciences, Nagasaki University, Nagasaki, Japan

**Keywords:** *Brachypodium distachyon*, cell wall fortification, phytohormone, quantitative resistance, *Rhizoctonia solani*, root infection

## Abstract

*Rhizoctonia solani* is a soil-borne phytopathogen with a broad host range, classified into multiple anastomosis groups (AGs). While the rice sheath blight pathogen AG-1 IA is well-studied, defense mechanisms against AG-4, which infects diverse monocots and dicots, remain unclear. Here, we screened 153 *Brachypodium distachyon* accessions for resistance to AG-4 HG-I+II using both leaf and soil inoculation assays. The accessions exhibited a quantitative resistance spectrum in both assays. Some accessions were resistant to both methods, while others showed resistance in only one, indicating that leaf and root defenses operate independently. Unlike the salicylic acid (SA)-dependent resistance observed in several accessions against AG-1 IA, AG-4 leaf resistance appears to be largely SA-independent. Among 22 resistant accessions, only eight induced the SA marker *BdWRKY38*, whereas twelve induced the jasmonic acid (JA) marker *BdAOS*. Additionally, resistance in Bd3–1 was unaffected by expression of the SA hydroxylase NahG. These results are consistent with previous findings that exogenous application of SA, JA, or ethylene fails to confer AG-4 resistance. Instead, most AG-4-resistant accessions showed common upregulation of cell wall biosynthesis-related genes following leaf infection, suggesting that cell wall fortification may contribute to defense against AG-4. These findings establish *B. distachyon* as a valuable model for dissecting resistance mechanisms against *R. solani* AG-4.

## 1 Introduction

*Rhizoctonia solani* is a soil-borne necrotrophic fungus recognized as one of the most destructive pathogens affecting a broad spectrum of economically important crops worldwide, including rice, wheat, maize, and various legumes (Anderson, 1982; Ogoshi, 1987). The significant annual yield losses caused by this pathogen have a global impact, threatening food security and agricultural sustainability. *R. solani* is a species complex comprising different isolates that are taxonomically classified into thirteen anastomosis groups (AGs), which are defined by their compatibility in hyphal fusion reactions (Carling et al., 2002; Ogoshi, 1987). For instance, *R. solani* AG-1 IA is the causal agent of rice sheath blight (Lee and Rush, 1983; Zheng et al., 2013), while AG-4 is associated with necrotic diseases in a wide range of both monocot and dicot crops (Ben et al., 2022; Choupannejad et al., 2017; Ginn et al., 2023; Mahadevan et al., 2025). The pathogen’s life cycle is characterized by its ability to persist in soil as sclerotia, enabling long-term survival and complicating disease management (Papavizas and Davey, 1962). As a necrotrophic pathogen, *R. solani* employs a diverse array of cell wall-degrading enzymes (CWDEs), also referred to as carbohydrate-active enzymes (CAZymes). These include glycoside hydrolases, glycosyltransferases, polysaccharide lyases, carbohydrate esterases, carbohydrate-binding modules, and auxiliary activity enzymes (Cantarel et al., 2009; Lombard et al., 2014). Notably, the genomes of AG-1 IA and AG-4 Rhs4ca encode 223 and 109 CAZymes, respectively (Zheng et al., 2013; Zhang et al., 2021).

Infection of leaves of rice, barley, and a model monocot *Brachypodium distachyon* by AG-1 IA typically involves the formation of densely distributed appressorium-like structures known as infection cushions, through which hyphae penetrate host tissues (Kouzai et al., 2018; Mahadevan et al., 2025; Matsuura, 1986). In contrast, AG-4 HG-I+II produces sparsely scattered hyphal masses accompanied by underlying necrotic lesions in host cells (Mahadevan et al., 2025). Pretreatment with salicylic acid (SA) confers resistance to AG-1 IA but not to AG-4 HG-I+II, suggesting that AG-1 IA undergoes an initial biotrophic phase before switching to necrotrophy, whereas AG-4 HG-I+II operates exclusively as a necrotroph (Kouzai et al., 2018; Mahadevan et al., 2025). These observations imply that the *R. solani* species complex encompasses isolates with at least two distinct infection strategies.

To mitigate the agricultural impact of *R. solani*, genetic loci associated with resistance have been extensively explored, particularly in rice against sheath blight. Unlike resistance to biotrophic or hemibiotrophic pathogens, resistance to AG-1 IA in rice is generally quantitative, with most cultivars exhibiting susceptibility but varying degrees of partial resistance (Hashiba and Kobayashi, 1996). Under field conditions, yield loss caused by AG-1 IA is influenced by heading date, because late summer conditions (high temperature and humidity) are favorable for sheath blight development, and cultivars that reach the susceptible growth stage during this period tend to suffer more severe disease (Taguchi-Shiobara et al., 2013). Quantitative trait locus (QTL) analyses using crosses between resistant and susceptible cultivars have identified multiple resistance loci through experimental designs that minimize confounding effects on plant development (Channamallikarjuna et al., 2010; Li et al., 1995; Taguchi-Shiobara et al., 2013; Yadav et al., 2015). Genome-wide association studies (GWAS) have also uncovered numerous resistance loci, including *OsRSR1* and *OsRLCK5*, which contribute to sheath blight resistance in rice (Chen et al., 2019; Naveenkumar et al., 2023; Zhang et al., 2019a; Wang et al., 2021).

To further explore the molecular mechanisms of plant resistance against *R. solani*, we employed natural accessions of *B. distachyon* as an understudied resource (Ayliffe et al., 2013; Bragg et al., 2012; Ogasahara et al., 2022). In *B. distachyon*, we previously identified several accessions, such as Bd3-1, Gaz-4, and Tek-3, that exhibit relatively high resistance to AG-1 IA leaf inoculation. These accessions rapidly induced the SA-responsive gene *BdWRKY38*, and fungal growth was consequently delayed (Kouzai et al., 2018; 2020). Network analysis further revealed that *BdWRKY38* functions as a key hub in the transcriptional regulation of defense responses, and the resistance of Bd3–1 accession has been shown to be SA-dependent (Kouzai et al., 2018; 2020).

In contrast, the characterization of resistance traits against AG-4 has not progressed as extensively as that against AG-1. Recently, 320 cultivars of buckwheat (*Fagopyrum tataricum*) were analyzed by genome-wide association studies (GWAS) for resistance to AG-4 HG-I 3, identifying 16 genomic regions containing 790 genes (He et al., 2023). Among these, 49 genes were upregulated in response to *R. solani* infection and methyl jasmonate treatment, and transient expression of five selected genes conferred *R. solani* resistance in *Arabidopsis*. One of these, an aspartic proteinase homolog gene (*FtASP*), encodes an antibacterial peptide that suppresses *R. solani* growth *in vitro*. These findings demonstrate the potential for developing AG-4 resistant crops. For future progress in molecular breeding, it is essential to elucidate the underlying resistance mechanisms and the genes involved across diverse plant species. In particular, resistance traits associated with *R. solani* infection of underground tissues remain poorly understood.

In this study, we investigated the resistance traits of *B. distachyon* against *R. solani* AG-4 HG-I+II using 153 accessions. Phenotypic screening in both leaf and soil inoculation assays revealed the quantitative nature of *B. distachyon* resistance to AG-4 in both tissues. Interestingly, resistance to AG-4 in these tissues appears to be an independent trait. Microscopic observation showed that AG-4 exhibits similar infection behavior and does not produce infection cushions on host leaves regardless of resistance level. The resistance of Bd3–1 to AG-1 IA is SA-dependent, whereas resistance to AG-4 is SA-independent. Consistently, defense marker genes for the SA, JA, and ET pathways were not commonly induced across the 22 accessions resistant to AG-4 leaf inoculation. Instead, we found that most of these resistant accessions tended to upregulate genes associated with cell wall fortification, pointing to a potential alternative defense strategy.

## 2 Materials and methods

### 2.1 Plant materials and growth conditions

Seeds of 153 genetically diverse *B. distachyon* accessions were obtained from the RIKEN Center for Sustainable Resource Science, Japan. The seeds were sterilized with 1% sodium hypochlorite for 5 min, rinsed three times with distilled water for 1 min each, and placed in Petri dishes lined with moist filter paper. The dishes were covered with aluminum foil and kept at 4 °C for three days for stratification. The seeds were then transferred to a growth chamber set at 23 °C with a 16 h/8 h photoperiod and a light intensity of 76 µmol m^-2^s^-1^ (BiOTRON NKsystem LH-411PFD, Osaka, Japan). Germinated seedlings were transplanted into 7-cm-diameter plastic pots filled with sterilized soil (Sakata Prime Mix TKS-1, Kanagawa, Japan) and grown under the same conditions. Accession Bd21, which is susceptible to both *R. solani* AG-1 IA and AG-4 HG-I+II, was used as a control throughout the experiments.

### 2.2 Fungal isolate and culture conditions

*R. solani* AG-4 HG-I+II (MAFF 305225), originally isolated from cauliflower, was obtained from the Genebank of the National Agriculture and Food Research Organization (NARO), Japan. The isolate was maintained by subculturing on potato dextrose agar (PDA; BD Difco™, Franklin Lakes, NJ, USA) and incubating the plates at 23 °C.

### 2.3 Root inoculation assay

The soil inoculation assay followed the method described by Mahadevan et al. (2025). Horticultural-grade vermiculite (100 g) (Uganda No. 3, AICHI-mederu, Nishio, Japan) and wheat bran (50 g) (Nisshin Seifun Welna, Tokyo, Japan) were mixed in a stainless-steel container with 300 mL of distilled water. After autoclaving, ten mycelial plugs (φ 8 mm) excised from a 3-day-old, actively growing *R. solani* AG-4 HG-I+II culture plate were added, and the mixture was incubated at 23 °C until the substrate was fully colonized. The colonized substrate was then mixed with autoclaved soil in a 1:4 (w/w) ratio and used to fill plastic pots, with three biological replicates per accession. Soil without inoculum served as the control. The pots were kept in the growth chamber, and disease severity was evaluated by measuring plant height daily for up to 7 days post-inoculation. Percentage growth retardation was calculated by the following equation:


$$Growth\;retardation\;\%=(\frac{Height\;of\;uninoculated\;plant-Height\;of\;inoculated\;plant\;}{Height\;of\;uninoculated\;plant})x\;100$$

### 2.4 Leaf inoculation assay

The leaf inoculation assay was performed as described by Kouzai et al. (2018). Briefly, the second newly emerged leaves of 3-week-old *B. distachyon* plants were detached and placed on moist filter paper in Petri dishes (φ 9 cm). A mycelial plug (φ 3 mm) excised from the edge of an *R. solani* AG-4 HG-I+II culture plate using a biopsy punch (BP-30F, Kai Corporation, Tokyo, Japan) was placed at the center of each detached leaf. A PDA plug without mycelium was used as an uninoculated control. The inoculated leaves were incubated in a growth chamber under high-humidity conditions for three days, after which symptoms were observed daily. Leaves were photographed, and the percentage of lesion area was quantified using Leaf Doctor software version 1.1 (Pethybridge and Nelson, 2015). Data from 3 days post-inoculation (dpi) were used to categorize resistance levels. A minimum of three biological replicates was used for each evaluation.

### 2.5 Fungal biomass quantification

Fungal biomass was measured as described by Kouzai et al. (2018). Inoculated leaf samples were snap-frozen in liquid nitrogen, and the tissues were homogenized with four zirconia balls (φ 3 mm) using a MicroSmash MS-100 homogenizer (TOMY SEIKO, Tokyo, Japan). Genomic DNA was extracted using the NucleoSpin Plant II kit (Takara Bio, Kusatsu, Japan), and DNA concentration and quality were assessed using a DS-11 spectrophotometer (DeNovix, Wilmington, DE, USA). Fungal biomass was quantified by real-time PCR using Luna^®^ Universal qPCR Master Mix (NEB, Ipswich, MA, USA) on a LightCycler^®^ 96 Real-Time PCR System (Roche, Basel, Switzerland). Primers specific for AG-4 detection (Budge et al., 2009) and the host reference gene *BdFIM4* for normalization (Zhu et al., 2014) were used (**Supplementary Table 3**).

### 2.6 RNA extraction and gene expression analysis

Inoculated *B. distachyon* leaves were collected at 6, 12, 24, and 48 hours post-inoculation (hpi). Aerial hyphae were removed using a 70% ethanol-moistened wipe, and the leaves were immediately frozen in liquid nitrogen and stored at −80 °C. Total RNA was extracted from each ground sample using the ISOSPIN Plant RNA kit (NIPPON GENE, Tokyo, Japan) following the manufacturer’s instructions. RNA concentration and purity were assessed using a spectrophotometer. cDNA was synthesized using the PrimeScript™ RT Reagent Kit with gDNA Eraser (Takara Bio). Quantitative real-time PCR was performed with Luna^®^ Universal qPCR Master Mix (New England Biolabs) on a LightCycler^®^ 96 Real-Time PCR System (Roche). Primers for phytohormone marker genes and cell wall biosynthesis genes are listed in **Supplementary Table 3** (Kouzai et al., 2016). Gene expression values were normalized to *BdUbi4* (*Bradi3g04730*) (Chambers et al., 2012). All experiments were performed with three biological replicates.

### 2.7 Microscopic observation

Inoculated leaves with adhering mycelia were collected at 3 dpi and placed in 2-mL tubes. The leaves were fixed using a graded ethanol series and incubated at 4 °C for three days to ensure complete chlorophyll removal. They were then stained overnight with 0.1% (w/v) lactophenol trypan blue, and excess stain was removed by incubating the samples in a chloral hydrate solution (2.5 g/mL) until the tissue became fully transparent. The cleared leaves were mounted on microscope slides and examined under a compact stereo microscope (ZEISS Stemi 305, Oberkochen, Germany).

### 2.8 Statistical analysis

Data were analyzed using SAS statistical software (PC-SAS 9.4 System, SAS Institute Inc., Cary, NC, USA). Experiments consisted of three biological replicates, and results are presented as mean ± SD. Statistical significance was assessed using Tukey’s HSD test or Student’s *t*-test.

## 3 Results

### 3.1 Leaf and root resistance of *B. distachyon* accessions to *R. solani* AG-4 HG-I+II

**Figure 1:**
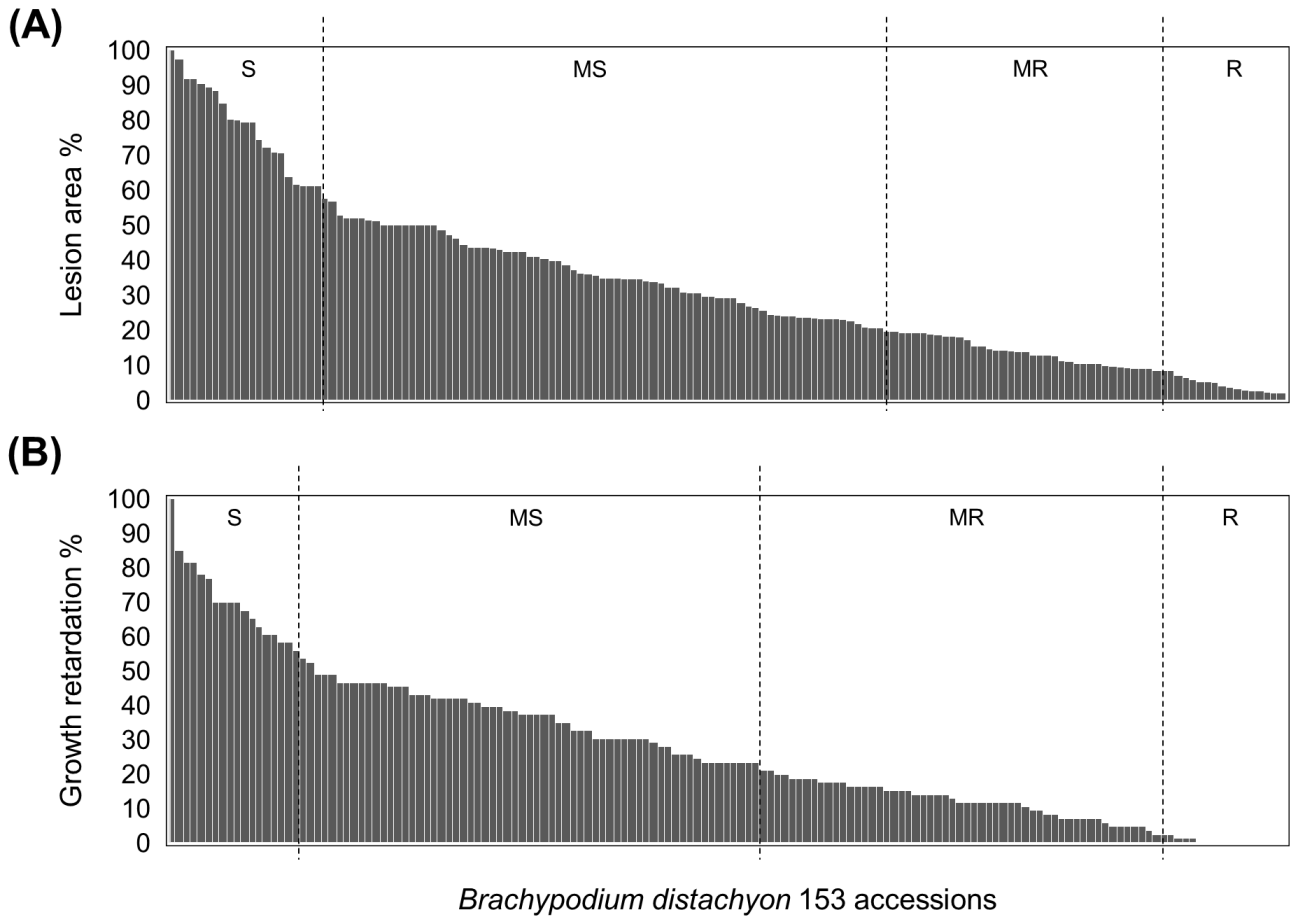
Phenotypic evaluation of disease resistance in *Brachypodium distachyon* accessions following leaf and soil inoculation with *Rhizoctonia solani* AG-4 HG I+II. **(A)** Disease resistance levels of *B. distachyon* accessions to leaf inoculation with AG-4 HG-I+II evaluated by lesion area measured at 3 days post-inoculation. **(B)** Disease resistance levels of *B. distachyon* accessions to soil inoculation with AG-4 HG-I+II evaluated by percent growth retardation measured at 7 days post-inoculation.

**Figure 2:**
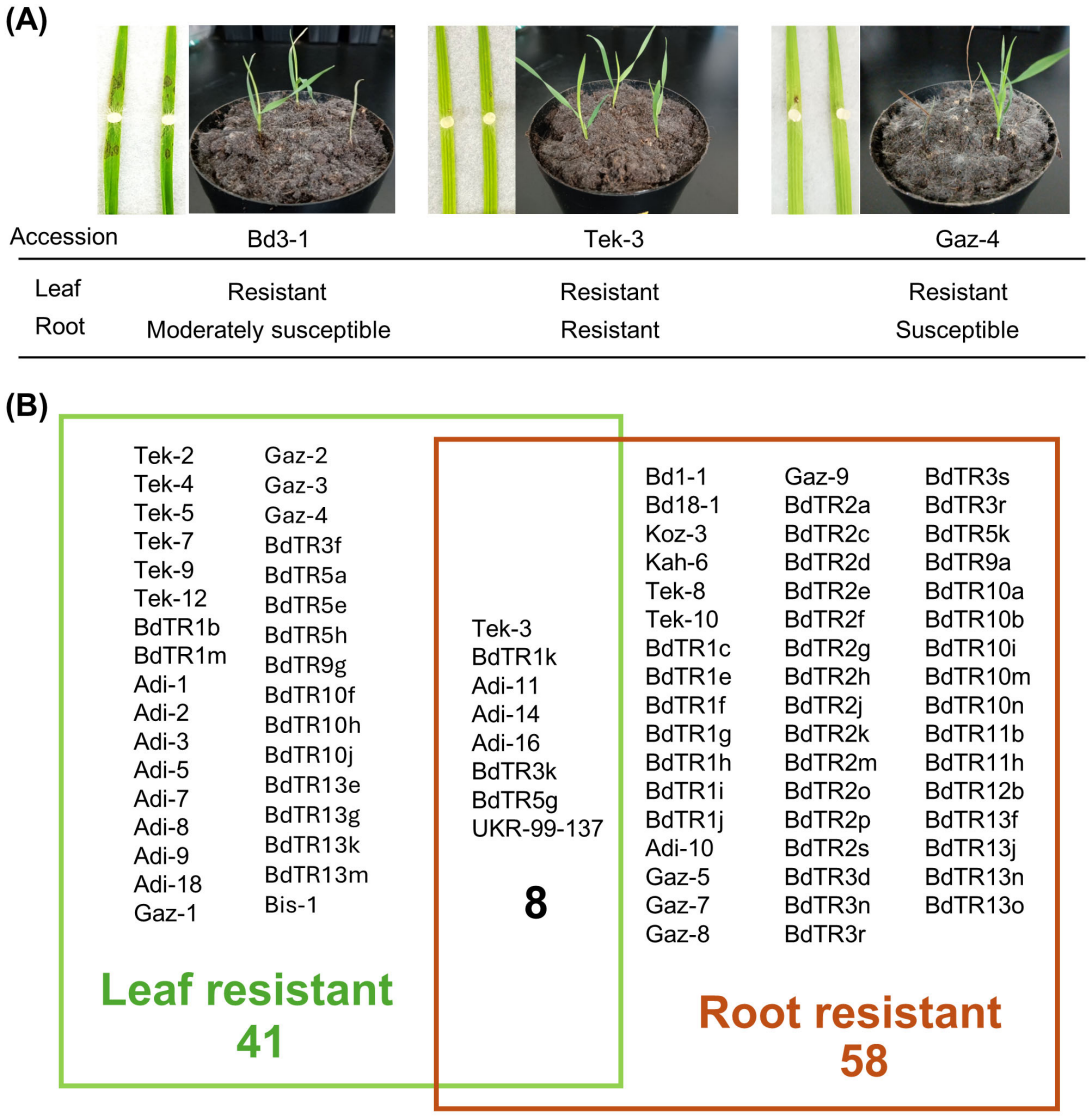
*Brachypodium distachyon* accessions showing resistance to leaf and soil inoculation with *Rhizoctonia solani* AG-4 HG-I+II. **(A)** Comparison of disease resistance in three representative *B. distachyon* accessions (Bd3-1, Tek-3, and Gaz-4) following leaf and soil inoculation with AG-4 HG I+II. Photographs were taken at 7 days post-inoculation (dpi) for soil assay and at 3 dpi for leaf assay. **(B)** List of *B. distachyon* accessions showing resistance to leaf and soil inoculation assays. Eight accessions exhibited resistance to both inoculation methods.

*B. distachyon* accessions collected across their natural range exhibit broad genetic diversity (Gordon et al., 2014; Vogel et al., 2009). To investigate resistance traits to AG-4 HG-I+II, we inoculated 153 accessions using leaf and soil inoculation assays. In the leaf assay, disease severity was quantified as the percentage of lesion area at 3 dpi. In the soil assay, disease severity was assessed by measuring plant height at 7 dpi. Because the standard accession Bd21 is known to be susceptible to AG-4 HG-I+II (Mahadevan et al., 2025), it was included in every experimental batch, and inter-batch variation was normalized relative to Bd21 results. The *B. distachyon* accessions displayed quantitatively distributed resistance in both leaf (**Figure 1A**; **Supplementary Table 1**) and root (**Figure 1B**; **Supplementary Table 2**) inoculation assays with AG-4 HG-I+II. Based on lesion area and growth retardation, accessions were classified into four categories for each assay: resistant (R), moderately resistant (MR), moderately susceptible (MS), and susceptible (S) (**Figure 1**; **Supplementary Figure 1**). Accessions exhibiting more than 50% and 25% lesion area or growth retardation were categorized as S and MS, respectively, whereas those displaying less than 25% and 15% were categorized as MR and R, respectively. The numbers of accessions in each category were 41, 30, 46, and 36 for the leaf assay and 58, 42, 26, and 27 for the root assay, respectively. A heatmap summarizing the resistance patterns of each accession for both assays is shown in **Supplementary Figure 2**. As reported previously (Mahadevan et al., 2025), Bd3-1, Tek-3, and Gaz-4 reproducibly showed leaf resistance, whereas Bd3–1 and Gaz-4 exhibited moderate susceptibility and susceptibility, respectively, in the root inoculation assay (**Figure 2A**; **Supplementary Figure 2**). The observed variation in resistance to AG-4 HG-I+II among the 153 accessions suggests that *B. distachyon* harbors diverse defense strategies against this pathogen.

Eight accessions (Tek-3, BdTR1k, Adi-11, Adi-14, Adi-16, BdTR3k, BdTR5g, and UKR-99-137) displayed resistance in both root and leaf inoculation assays (**Figure 2B**). In contrast, 33 accessions showed resistance only in the leaf assay (Tek-2, Tek-4, Tek-5, Tek-7, Tek-9, Tek-12, BdTR1b, BdTR1m, Adi-1, Adi-2, Adi-3, Adi-5, Adi-7, Adi-8, Adi-9, Adi-18, Gaz-1, Gaz-2, Gaz-3, Gaz-4, BdTR3f, BdTR5a, BdTR5e, BdTR5h, BdTR9g, BdTR10f, BdTR10h, BdTR10j, BdTR13e, BdTR13g, BdTR13k, BdTR13m, and Bis-1), whereas 50 accessions showed resistance only in the root assay (Bd1-1, Bd18-1, Koz-3, Kah-6, Tek-8, Tek-10, BdTR1c, BdTR1e, BdTR1f, BdTR1g, BdTR1h, BdTR1i, BdTR1j, Adi-10, Gaz-5, Gaz-7, Gaz-8, Gaz-9, BdTR2a, BdTR2c, BdTR2d, BdTR2e, BdTR2f, BdTR2g, BdTR2h, BdTR2j, BdTR2k, BdTR2m, BdTR2o, BdTR2p, BdTR2s, BdTR3d, BdTR3n, BdTR3r, BdTR3s, BdTR3t, BdTR5k, BdTR9a, BdTR10a, BdTR10b, BdTR10i, BdTR10m, BdTR10n, BdTR11b, BdTR11h, BdTR12b, BdTR13f, BdTR13j, BdTR13n, and BdTR13o) (**Figures 1A, B**, **2B**). Of the three accessions previously reported to exhibit leaf resistance to AG-4 HG-I+II (Bd3-1, Tek-3, and Gaz-4), only Tek-3 showed resistance in the root assay, whereas Bd3–1 was moderately susceptible and Gaz-4 was susceptible (**Figure 2B**). These results indicate that resistance to AG-4 HG-I+II in *B. distachyon* is tissue-specific. Although most accessions used in this study originate from Turkey, with seven additional accessions from Iraq, Spain, and Ukraine, the distribution of leaf and root resistance traits does not appear to correlate with evolutionary or geographical characteristics based on the previously reported phylogenetic relationships of *B. distachyon* accessions (Vogel et al., 2009).

### 3.2 Phytohormone-related responses in leaf-resistant *B. distachyon* accessions

**Figure 3:**
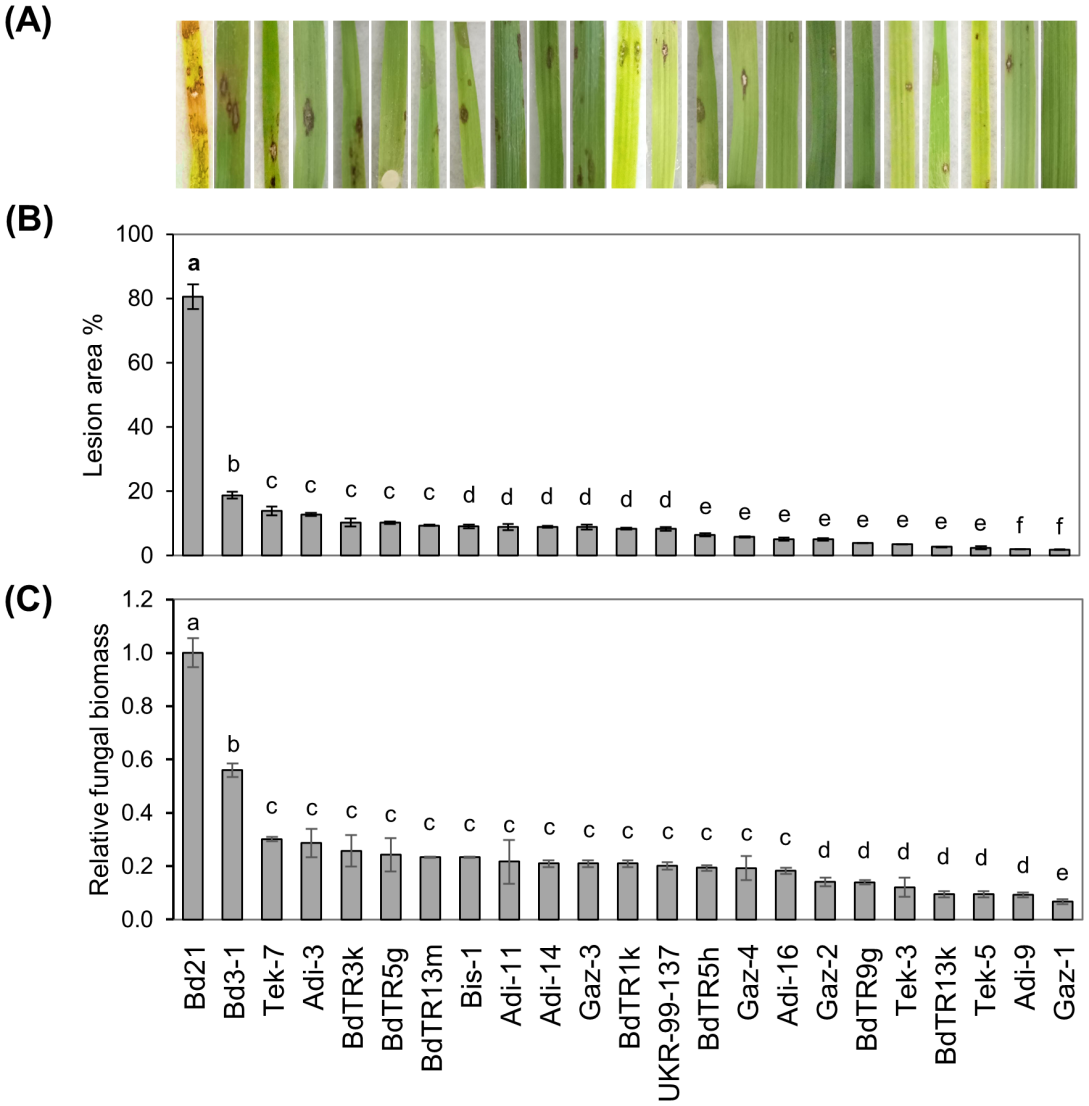
Phenotypic comparison of *Brachypodium distachyon* accessions exhibiting resistance to leaf inoculation with *Rhizoctonia solani* AG-4 HG-I+II. **(A–C)** Disease symptoms **(A)**, percent lesion area **(B)**, and relative fungal biomass **(C)** in inoculated leaves of the top 22 *B. distachyon* accessions resistant to AG-4 HG-I+II. Lesion areas were analyzed from photographs taken at 3 days post-inoculation, and the same leaves were subsequently used for fungal biomass measurement. Data are presented as mean values ± SD (*n* = 3). Different letters indicate statistically significant differences (Tukey’s HSD test, *P* < 0.05).

From the 41 accessions showing resistance in the leaf inoculation assay, we selected 22 in which symptoms did not appear until 3 dpi for further investigation of their defense strategies. Representative symptoms at 3 dpi are shown in **Figure 3A**, and percentage of lesion area is presented in **Figure 3B**. All selected accessions exhibited less than 20% necrotic lesion area compared with the susceptible control accession Bd21. Fungal biomass was also quantified by qPCR (**Figure 3C**), and the extent of colonization generally correlated with lesion area. These results confirm the resistant phenotypes of the selected accessions and suggest that resistance may be attributed to restricted pathogen colonization.

**Figure 4:**
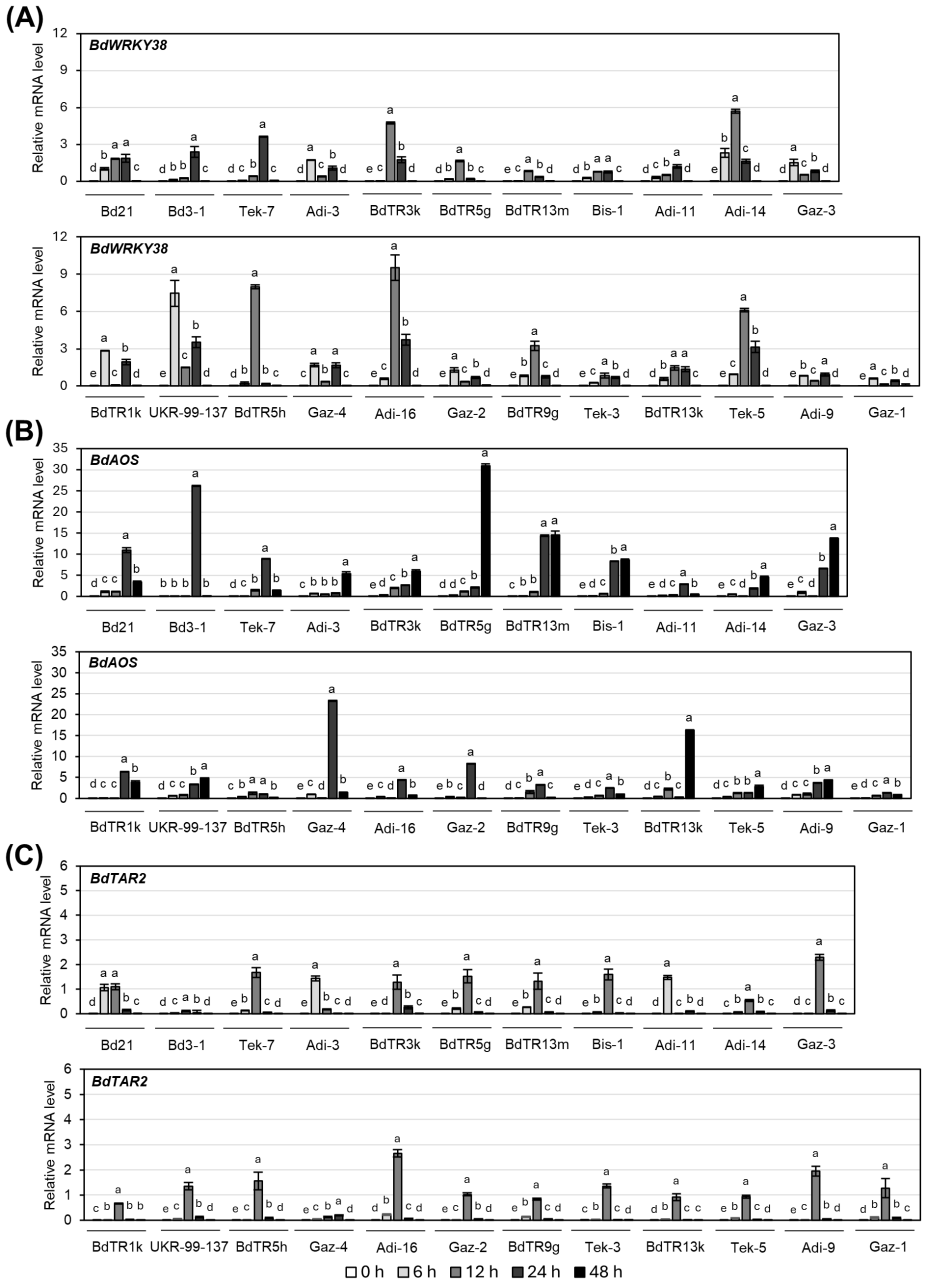
Expression profiles of phytohormone-related genes in 22 leaf-resistant *Brachypodium distachyon* accessions during *Rhizoctonia solani* AG-4 HG-I+II infection. **(A–C)** Relative mRNA levels of *BdWRKY38*, a salicylic acid-responsive marker gene **(A)**, *BdAOS*, a jasmonic acid-responsive marker gene **(B)**, and *BdTAR2*, an ethylene-responsive marker gene **(C)**, in leaves of *B. distachyon* accessions at 6, 12, 24 and 48 hours post-inoculation with AG-4 HG-I+II. Bd21 was used as a representative susceptible accession. Data are presented as mean values ± SD (*n* = 3). Different letters indicate statistically significant differences among time points within each accession (one-way ANOVA followed by Tukey’s HSD *post hoc* test, *P* < 0.05).

Previous studies have shown that phytohormone-mediated signaling plays a key roles in regulating plant resistance to *R. solani* (He et al., 2023; Kidd et al., 2021; Koley et al., 2022; Kouzai et al., 2018; Liu et al., 2017; Mahadevan et al., 2025). To determine whether SA-, JA-, or ET-related pathways were activated in the selected resistant accessions during AG-4 HG-I+II infection, we examined the expression of *BdWRKY38* (WRKY-type transcription factor; SA marker), *BdAOS* (allene oxide synthase; JA marker), and *BdTAR2* (tryptophan aminotransferase; ET marker) as markers at 6, 12, 24, and 48 hpi (Kouzai et al., 2016). Only eight accessions (Tek-7, BdTR3k, Adi-14, UKR-99-137, BdTR5h, Adi-16, BdTR9g, and Tek-5) showed more than 3-fold induction of *BdWRKY38* at any time point (**Figure 4A**). *BdAOS* was induced more than 5-fold in Bd21, Bd3-1, Tek-7, BdTR13m, Bis-1, Gaz-3, BdTR1k, Gaz-4, and Gaz-2 at 24 hpi, whereas similar induction was observed in Adi-3, BdTR3k, BdTR5g, BdTR13m, Bis-1, Gaz-3, and BdTR13k at 48 hpi (**Figure 4B**). Interestingly, the accessions inducing SA- or JA-responsive marker genes at 24 hpi showed little overlap, with the exception of Tek-7. Expression of *BdTAR2* in the resistant accessions did not differ significantly from that in Bd21 (**Figure 4C**). These results suggest that at least two independent defense mechanisms, one SA-dependent and the other JA-dependent, may contribute to resistance against AG-4 HG-I+II. However, several accessions, including Adi-11, Tek-3, Adi-9, and Gaz-1, did not induce these marker genes according to the above-mentioned criteria.

**Figure 5:**
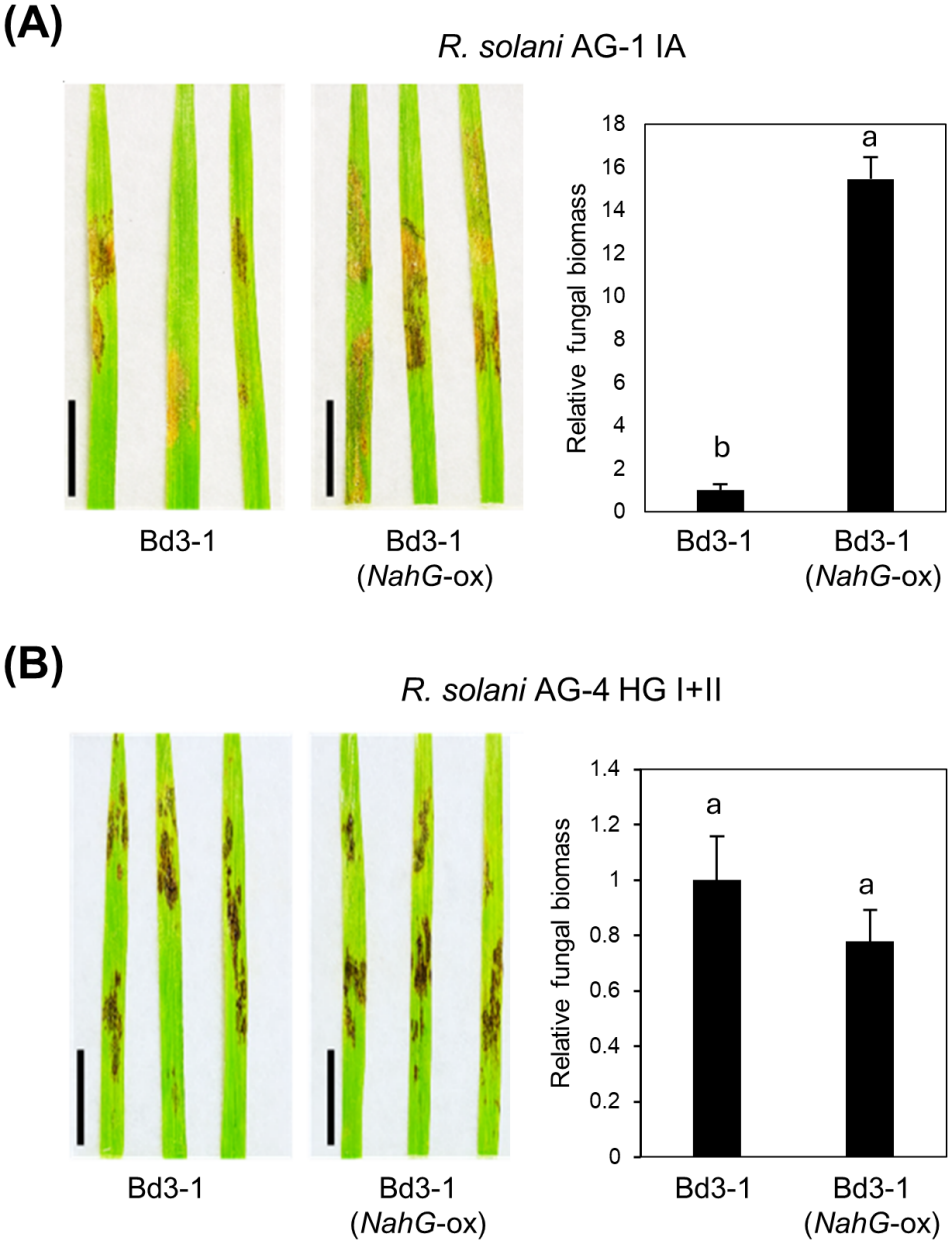
Phenotypic comparison of wild-type and *NahG*-expressing *Brachypodium distachyon* Bd3–1 in response to inoculation with *Rhizoctonia solani* AG-1 IA and AG-4 HG-I+II. **(A, B)** Lesion formation and relative fungal biomass in wild-type and *NahG*-overexpressing Bd3–1 leaves inoculated with AG-1 IA **(A)** and AG-4 HG-I+II **(B)** at 3 days post-inoculation. Line #1 of the *NahG* transgenic plants described by Kouzai et al. (2020) was used in this study. Data are presented as mean values ± SD (*n* = 3). Different letters indicate statistically significant differences (one-way ANOVA followed by Tukey’s HSD *post hoc* test, *P* < 0.05).

To further examine the involvement of SA in resistance to AG-4 HG-I+II, we used Bd3–1 plants overexpressing *NahG*. As reported previously (Kouzai et al., 2020), lesion area and fungal biomass increased in *NahG*-expressing Bd3–1 plants after AG-1 IA infection (**Figure 5A**). In contrast, Bd3–1 exhibited a similar level of resistance to AG-4 HG-I+II regardless of *NahG* expression (**Figure 5B**). This indicates that SA does not contribute to Bd3–1 resistance against AG-4 HG-I+II, although *BdWRKY38* showed slight induction at 24 hpi. These findings are also consistent with earlier results showing that pretreatment with SA or *N*-hydroxypipecolic acid (NHP) confers resistance to AG-1 IA but not to AG-4 HG-I+II in Bd21 (Mahadevan et al., 2025).

### 3.3 Mycelial growth of AG-4 HG-I+II and symptoms in resistant *B. distachyon* accessions

**Figure 6:**
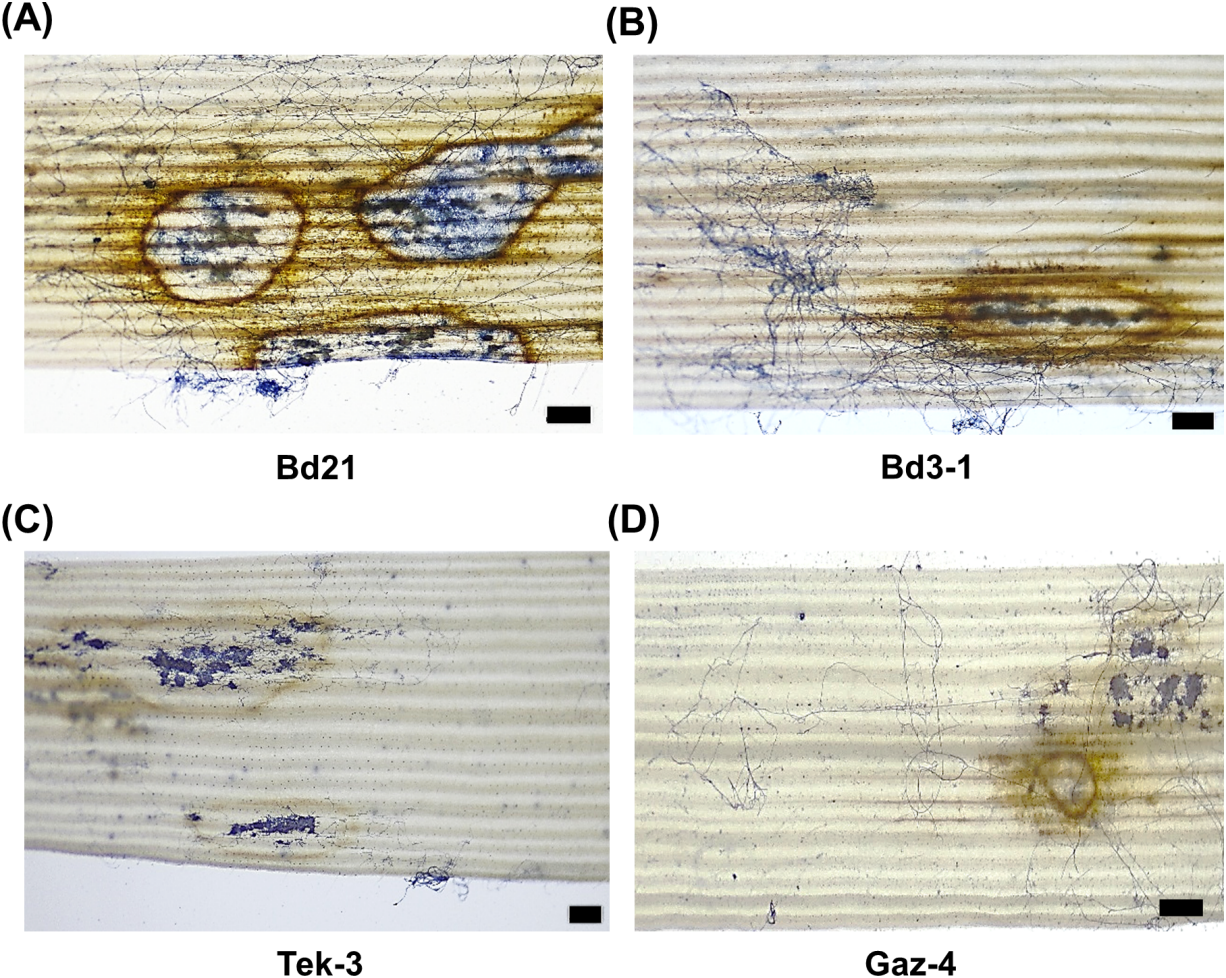
Trypan blue staining of inoculated leaves from selected resistant *Brachypodium distachyon* accessions following *Rhizoctonia solani* AG-4 HG-I+II infection. **(A–D)** Fungal hyphae and lesions stained with trypan blue in leaves of susceptible Bd21 **(A)**, and resistant Bd3-1 **(B)**, Tek-3 **(C)**, and Gaz-4 **(D)** at 3 days post-inoculation with AG-4 HG-I+II. Brown discoloration was observed surrounding the necrotic lesions. Bar, 1 mm.

To further characterize the defense mechanisms, we examined the hyphal growth of AG-4 HG-I+II on the leaf surface of resistant *B. distachyon* accessions. As reported previously (Mahadevan et al., 2025), AG-4 HG-I+II produced scattered mycelial masses on the leaves of susceptible Bd21 at 3 dpi, accompanied by expanding necrotic lesions beneath them (**Figure 6A**). In this study, we monitored hyphal development on the resistant accessions Bd3-1, Tek-3, and Gaz-4. Consistent with the reduced fungal biomass observed in these accessions (**Figure 3C**), both the amount and density of hyphae were markedly lower on their leaf surfaces (**Figures 6B–D**). These observations suggest that resistant accessions may suppress fungal proliferation, potentially through antimicrobial activity at the leaf surface. Closer inspection also revealed reduced pigment accumulation around necrotic areas in the resistant accessions. Taken together, these results suggest that these accessions may limit the effectiveness of pathogen-secreted CWDEs and CAZymes, thereby constraining fungal colonization and nutrient acquisition even in regions where mycelial masses have formed.

### 3.4 Expression of cell wall biosynthesis genes in resistant *B. distachyon* accessions

**Figure 7:**
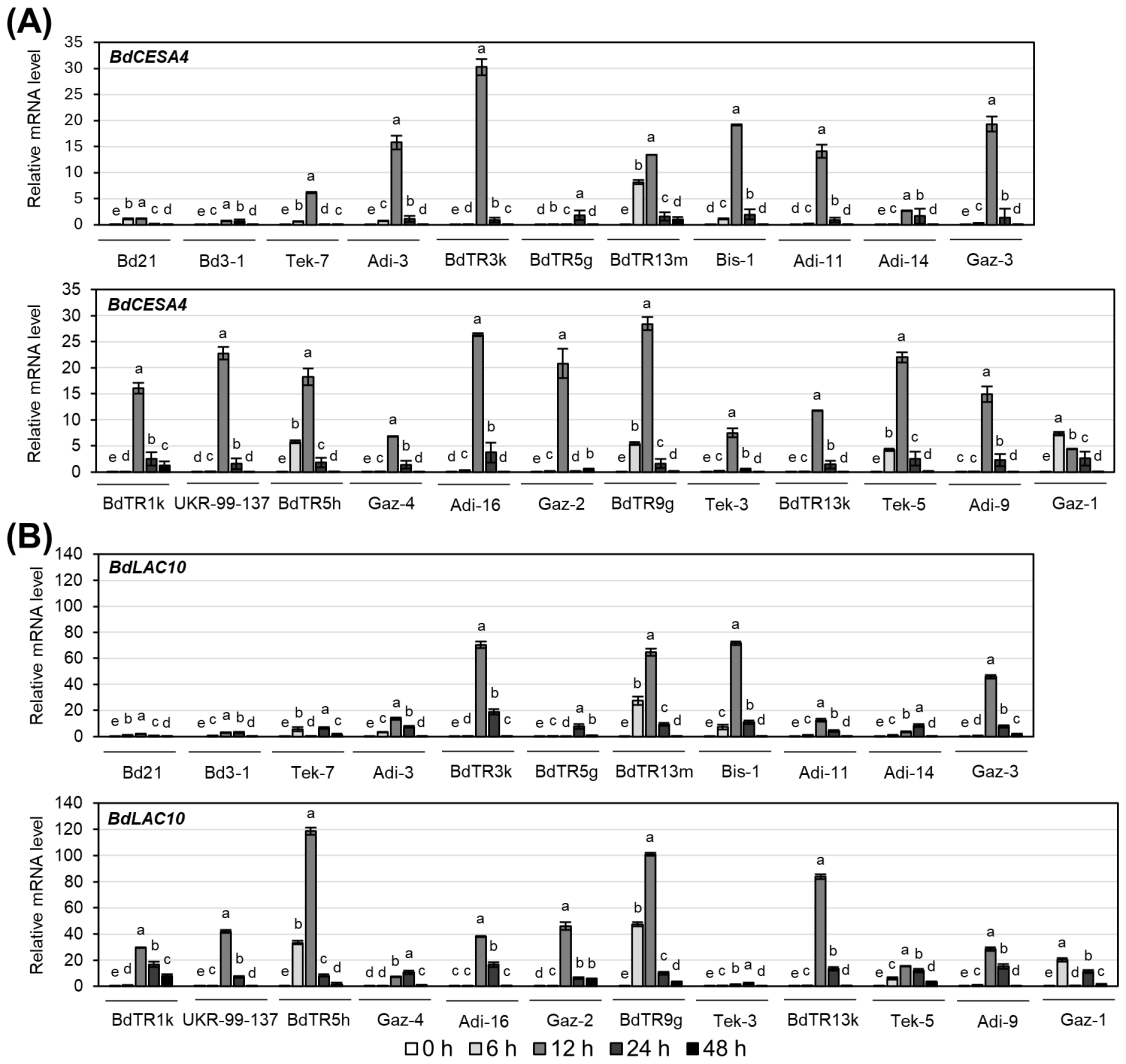
Expression profiles of cell wall biosynthesis-related genes in 22 leaf-resistant *Brachypodium distachyon* accessions during *Rhizoctonia solani* AG-4 HG-I+II infection. **(A, B)** Relative mRNA levels of *BdCESA4* (*cellulose synthase A*), a marker gene for cell wall cellulose biosynthesis **(A)** and *BdLAC10* (*laccase 10*), a marker gene for lignin biosynthesis **(B)**, in leaves of *B. distachyon* accessions at 6, 12, 24 and 48 hours post-inoculation with AG-4 HG-I+II. Bd21 was used as a representative susceptible accession. Data are presented as mean values ± SD (*n* = 3). Different letters indicate statistically significant differences among time points within each accession (one-way ANOVA followed by Tukey’s HSD *post hoc* test, *P* < 0.05).

In plants, cell wall fortification through lignification and polysaccharide modification is a well-established defense response to pathogen attack (Coomey et al., 2020; Ishida and Noutoshi, 2022). To determine whether cell wall reinforcement contributes to *B. distachyon* resistance to AG-4 HG-I+II, we analyzed the expression of two cell wall biosynthesis-related genes, *BdCESA4* and *BdLAC10*, at 6, 12, 24, and 48 hpi (**Figure 7**). Cellulose synthase A (CESA) genes are responsible for cellulose biosynthesis, with *BdCESA4* specifically involved in secondary cell wall (SCW) formation (Handakumbura et al., 2013; Hernández-Blanco et al., 2007; Yang et al., 2021). *BdLAC10*, a member of the laccase family, is associated with lignin accumulation (Wang et al., 2015). Most accessions, except Bd3-1, BdTR5g, and Adi-14, showed more than 5-fold induction of *BdCESA4*, with some accessions reaching nearly 30-fold expression by 6 or 12 hpi. Similarly, *BdLAC10* expression was induced more than 10-fold (and up to approximately 120-fold) in most accessions, except Bd3-1, Tek-7, BdTR5g, Adi-14, and Tek-3, at early time points. Although induction levels were lower, *BdLAC10* expression was detectable in Tek-7, BdTR5g, and Adi-14. Collectively, most resistant accessions induced either *BdCESA4* or *BdLAC10* during AG-4 HG-I+II infection, suggesting that cell wall reinforcement may be associated with resistance-related responses in *B. distachyon* accessions. In this study, the expression of these genes was not detected in Bd3-1. Instead, this accession may induce homologous genes, such as *BdCESA7*, *BdCESA8*, and *BdLAC6* (Kouzai et al., 2018); alternatively, preformed defense mechanisms may play a major role in its resistance.

### 3.5 Infection behavior of AG-4 HG-I+II in susceptible *B. distachyon* accessions

Both AG-1 IA and AG-4 HG-I+II infect Bd21 and produce necrotic lesions; however, AG-1 IA forms densely distributed infection cushions, whereas AG-4 HG-I+II generates dispersed hyphal masses (Kouzai et al., 2018; 2020; Mahadevan et al., 2025). The same isolate of AG-1 IA can infect Arabidopsis leaves, but in this host its infection is not suppressed by SA pretreatment, even though SA inhibits AG-1 IA infection in *B. distachyon* (Abdelghany et al., 2022). These findings suggest that *R. solani* isolates may modulate their infection strategy depending on the host species or host condition.

To further evaluate this possibility, we examined the infection hyphae of AG-4 HG-I+II on susceptible *B. distachyon* accessions. Among the accessions classified as susceptible (S) based on lesion area (**Figure 1**; **Supplementary Figure 2**), BdTR2h, Koz-3, BdTR10m, X4006, and Adi-4 were selected for further observation, as they developed symptoms as early as 18 hours post-inoculation (hpi). In addition, Bd21 was included as a susceptible control, with symptom onset occurring at 24 hpi. The hyphae extending on the leaves of these accessions appeared similar to those observed on Bd21 (**Supplementary Figure 3**) and also comparable to those on resistant accessions (**Figure 6**). These results suggest that AG-4 HG-I+II employs essentially the same infection strategy on *B. distachyon* regardless of host resistance level. Although some susceptible accessions showed weak induction of *BdWRKY38*, expression levels remained below 5-fold (**Supplementary Figure 4A**). All of these susceptible accessions, however, commonly induced *BdAOS* at 24 hpi (**Supplementary Figure 4B**), which may reflect a JA-mediated wounding response triggered by lesion formation. No significant induction of *BdTAR2*, an ET-responsive gene, was observed (**Supplementary Figure 4C**). In contrast to the resistant accessions, which strongly induced the cell wall-related genes *BdCESA4* and *BdLAC10* (particularly at 12 hpi), these genes were not markedly induced in the susceptible accessions (**Supplementary Figures 5A, B**). Together, these results suggest a potential association between the induction of cell wall fortification-related responses and resistance to AG-4 HG-I+II in *B. distachyon* accessions.

## 4 Discussion

Because *R. solani* causes severe agricultural losses, a deeper understanding of its virulence mechanisms is essential for developing effective control strategies (Anderson, 1982; Ogoshi, 1987). However, progress in this area has been hampered by technical limitations, particularly the difficulty of performing gene knockout or knockdown through transformation in *R. solani*. Although heterologous expression of virulence proteins, including effectors, in host plants and host-induced gene silencing have recently facilitated functional studies (Dölfors et al., 2019; Ghag et al., 2014; Tzelepis et al., 2021; Zhang et al., 2022; 2024; Zhao et al., 2021; 2024; Zheng et al., 2013), these approaches remain indirect. Further complicating the issue is the nature of the *R. solani* species complex: virulence mechanisms and host specificity are not always correlated with AG classification (Abdelghany et al., 2022; Mahadevan et al., 2025; Pannecoucque and Höfte, 2009). Nevertheless, high-quality whole-genome datasets generated using long-read sequencing have revealed substantial genomic diversity within the species complex (Li et al., 2021; Nadarajah et al., 2017; Yang et al., 2022; Zhang et al., 2021). To address these challenges, model plants can serve as valuable platforms. Differences in host defense responses and pathogen infection behavior among genetically diverse plant accessions can provide important clues to virulence strategies. Using *B. distachyon*, we previously showed that AG-1 IA may employ a brief biotrophic phase during the early stages of infection (Kouzai et al., 2018; 2020). In addition, our comparisons of multiple *R. solani* isolates revealed that AG-4 HG-I+II is capable of infecting both leaves and roots of *B. distachyon* and barley, in contrast to AG-1 IA, which infects only the leaves of these species (Mahadevan et al., 2025).

In this study, we used a panel of *B. distachyon* accessions to further characterize variation in resistance to AG-4 HG-I+II. The accessions showed continuous variation in resistance in both leaf and root inoculation assays, consistent with the presence of diverse defense mechanisms. Accessions that were resistant in leaf assays were not necessarily resistant in root assays, suggesting that resistance in leaves and roots may be controlled, at least in part, by tissue-specific mechanisms rather than by a uniform whole-plant defense response. Such tissue specificity may arise from spatially regulated gene expression, differential activation of immune signaling pathways, and localized accumulation of defensive metabolites (Amin, 2026; Piasecka et al., 2015). For example, studies in maize have shown that roots can differ from leaves in basal levels and induction kinetics of antimicrobial compounds (Balmer et al., 2013), and defensive secondary metabolites can contribute to organ-specific resistance phenotypes in leaves, stems, and reproductive tissues (Balmer et al., 2013; Munch et al., 2018). Consistent with this concept, tissue-specific genetic loci have also been associated with leaf-specific, but not fruit-specific, resistance to strawberry powdery mildew (Lynn et al., 2024). These findings have important implications for future breeding and GWAS studies: resistance phenotypes should be evaluated separately in leaves and roots, and tissue-specific phenotypes may help identify loci that would otherwise be missed in whole-plant or single-tissue screens. For breeding purposes, combining resistance loci effective in different tissues may be necessary to develop germplasm with more robust resistance to AG-4 HG-I+II. In addition, the tissue-dependent resistance observed here raises the possibility that AG-4 HG-I+II deploys distinct virulence strategies in leaves and roots. Although visualization of infection processes in underground tissues remains technically challenging, limiting direct analysis of root virulence mechanisms, molecular characterization of plant defense responses to *R. solani* root infection will be an important direction for future research.

Among the 22 *B. distachyon* accessions that exhibit resistance to AG-4 HG-I+II in this study, eight clearly induced *BdWRKY38* and thirteen induced *BdAOS* during leaf infection, while four accessions showed no obvious induction of either gene. This variation likely reflects the diversity of defense responses present within *B. distachyon*. Because these resistant accessions developed little to no visible lesions after inoculation and showed strong induction of the marker genes, the observed JA responses are presumed to be actively triggered rather than passively induced by wounding. Thus, JA-mediated defense activation may contribute to resistance against AG-4 HG-I+II. Although pretreatment with SA, JA, or ET failed to confer detectable resistance to AG-4 HG-I+II in the susceptible accession Bd21, and although Bd3–1 resistance to AG-4 HG-I+II is SA-independent, it remains possible that induction of these hormone-mediated pathways contributes to resistance to some extent in specific accessions. For example, Bd3–1 induces *BdWRKY38* and shows SA-dependent resistance upon AG-1 IA infection (Kouzai et al., 2018; 2020). Because AG-1 IA upregulates several effector-like protein-encoding genes during infection (Abdelsalam et al., 2020), Bd3–1 may recognize pathogen-derived effectors and activate SA-mediated defense. Similarly, some AG-4 HG-I+II-resistant accessions may perceive effector-like molecules and mount an SA response. Two AG-4 HG-I isolates (Rs23 and R118-11) have been predicted to encode 122 and 133 effector-like proteins, respectively, comparable to other isolates, although it remains unclear whether these genes are expressed during infection (Kaushik et al., 2022). To evaluate this possibility, effector prediction using the AG-4 HG-I+II genome combined with expression profiling during infection will be required. In accessions that activate SA-related signaling, JA-dependent signaling may be suppressed. The presence of accessions that did not induce either the SA or JA marker genes suggests the involvement of additional defense processes, such as pattern-triggered immunity mediated by recognition of microbe-associated molecular patterns (MAMPs), in resistance to AG-4 HG-I+II.

In most of the resistant accessions examined, we observed strong induction of marker genes associated with cell wall biosynthesis. Based on these results, cell wall biogenesis-related responses may be associated with resistance to AG-4 HG-I+II infection. This interpretation is consistent with previous findings showing that mutations in secondary wall cellulose synthases (e.g., *CESA4*, *CESA7*, and *CESA8* in Arabidopsis) alter resistance to various pathogens (Hernández-Blanco et al., 2007). In rice, knockdown of *OsCESA4* disrupts *Xa4*-mediated resistance to *Xanthomonas oryzae* (Hu et al., 2017). Enhanced lignification also functions as a parallel defense strategy, strengthening the cell wall, creating hydrophobic barriers that hinder pathogen enzyme activity, and reducing access to wall polysaccharides (Ma, 2024). Lignification mediated by a cotton laccase contributes to resistance against Verticillium wilt (Zhang et al., 2019b). To further elucidate the structural basis of the observed resistance, quantitative assessment of cell wall composition, particularly lignin content, in the resistant and susceptible accessions identified in this study would provide important insights into the underlying resistance mechanisms. Lignin is widely recognized as a key component of plant defense because its deposition can reinforce the cell wall and limit pathogen entry and spread (Lee et al., 2019; Ma, 2024). Recent studies also indicate that cell wall remodeling is a major determinant of resistant and susceptible plant-pathogen interaction outcomes. For instance, increased lignin accumulation has been directly associated with resistance to *Plasmodiophora brassicae*, the causal agent of clubroot disease (Tu et al., 2024). While the pathogen can degrade cell wall lignin in the susceptible variety ‘Westar’, resistant accessions actively accumulate lignin around infection sites (Tu et al., 2024). This lignin deposition was accompanied by the upregulation of genes encoding enzymes involved in the phenylpropanoid pathway (Mutuku et al., 2019).

We previously demonstrated that SA-induced defense responses in Bd21 are significantly enriched in biological processes related to cell wall biogenesis (Kouzai et al., 2018); thus, the cell wall genes induced in some accessions may be regulated by SA-mediated signaling. Further studies are needed to determine whether JA or MAMP-triggered signaling also regulates these cell wall-related cellular processes. Although most resistant accessions induced either defense marker genes or cell wall biosynthesis genes, it remains possible that preformed defenses substantially contribute to resistance against AG-4 HG-I+II. This idea is supported by some accessions (Bd3-1, BdTR5g, and Adi-14) that showed either no or only weak induction of the tested cell wall biosynthesis genes during infection. It is also consistent with previous reports showing extensive natural variation in *B. distachyon* cell wall composition and biomass digestibility, with some compositional traits correlating with growth characteristics (Cass et al., 2016). In rice, for example, cultivars exhibiting higher constitutive expression of defense genes such as *PBZ1* show increased resistance to *Magnaporthe oryzae*, independent of SA, JA, or ET signaling (Vergne et al., 2010). However, our current data suggest that *B. distachyon* resistance to AG-4 HG-I+II may not primarily rely on constitutive defense activation.

We previously demonstrated that AG-1 IA and AG-4 HG-I+II exhibit distinct infection behaviors on both *B. distachyon* and barley leaves, despite both causing severe disease symptoms. This raises intriguing questions regarding the diversity and evolution of infection strategies within *R. solani*. One possibility is that AG-4 HG-I+II retains the genetic capacity to form infection cushions but does not deploy them on *B. distachyon* or barley leaves (Mahadevan et al., 2025). We hypothesized that AG-4 HG-I+II might alter its infection process depending on host conditions. However, examination of infection hyphae on both resistant and susceptible accessions revealed no apparent variation in infection structures, and we did not detect any condition-dependent changes in infection strategy under our experimental settings. To determine whether AG-4 HG-I+II indeed employs a single strategy regardless of host resistance, further studies involving additional host species combined with comparative genomics will be required.

The phenotypic analyses in this study suggest multiple possible defense strategies in *B. distachyon* against AG-4 HG-I+II, including hormone-mediated responses, cell wall fortification, and potential preformed barriers. However, because these observations were made using a detached-leaf assay, they may not fully capture the whole-plant physiological dynamics that occur during infection of intact plants. Although detached-leaf assays are useful for standardized screening, they may compromise some host defense responses and fail to reflect variation at the whole-plant level. In addition, detached- and attached-leaf assays can reveal distinct defense responses, with symptom development in detached tissues sometimes becoming uncoupled from canonical defense signaling and more closely associated with senescence-related processes. To minimize this effect, uninfected detached leaves were used as baseline controls to distinguish pathogen-specific responses from general responses caused by tissue detachment. Further studies using intact plants, together with the identification of host genes underlying quantitative resistance, will be needed to better understand the defense mechanisms involved in resistance to AG-4 HG-I+II. In this regard, genome-wide association studies (GWAS) using the 153 accessions are expected to provide high-resolution insights into the mechanisms of resistance to AG-4 HG-I+II, as well as contribute to a broader understanding of virulence diversification within the *R. solani* species complex.

## Data Availability

The original contributions presented in the study are included in the article/**Supplementary Material**. Further inquiries can be directed to the corresponding author/s.
