# Supplementary material for: Quantitative resistance to Rhizoctonia solani AG-4 develops independently in leaves and roots of Brachypodium distachyon
Source: Front Plant Sci. 2026 Jul 3;17:1875734. doi: 10.3389/fpls.2026.1875734 (PMC13375911; doi:10.3389/fpls.2026.1875734)
Supplement: Supplementary file 1 [file DataSheet1.pdf]

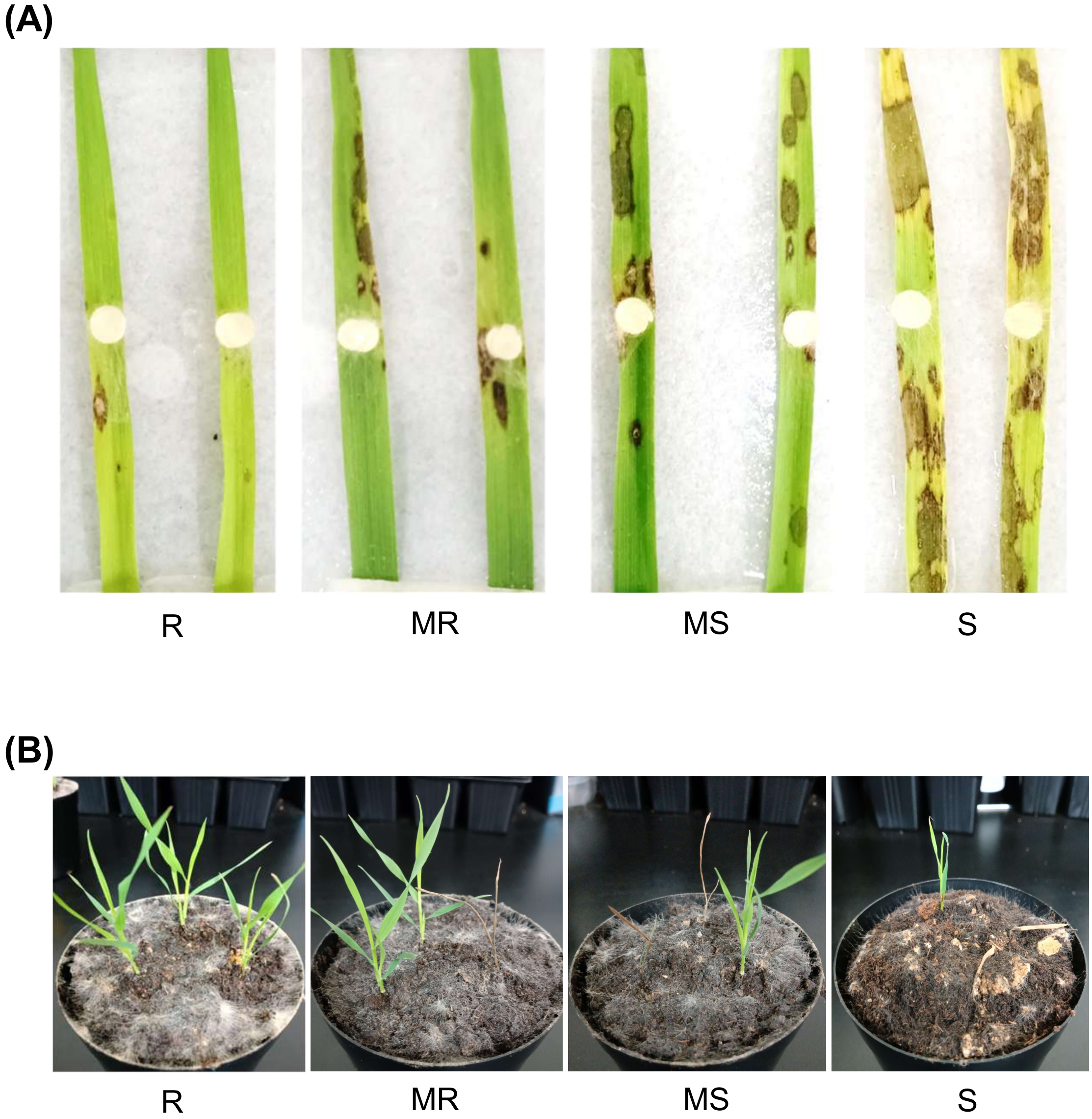

Supplementary Figure 1. Criteria for classifications of disease resistance levels in *Brachypodium distachyon* accessions following leaf and soil inoculation with *Rhizoctonia solani* AG-4 HG-I+II. **(A,B)** Examples of disease resistance classification for leaf (A) and soil (B) inoculation assays. For leaf inoculation, accessions were classified into four categories based on percent lesion area: resistant (R, <15%), moderately resistant (MR, 15–25%), moderately susceptible (MS, 25–50%), and susceptible (S, <50%). For soil inoculation, accessions were classified into four categories based on percent growth retardation: R (<15%), MR (15–25%), MS (25–50%), and S (<50%) compared to uninoculated controls.

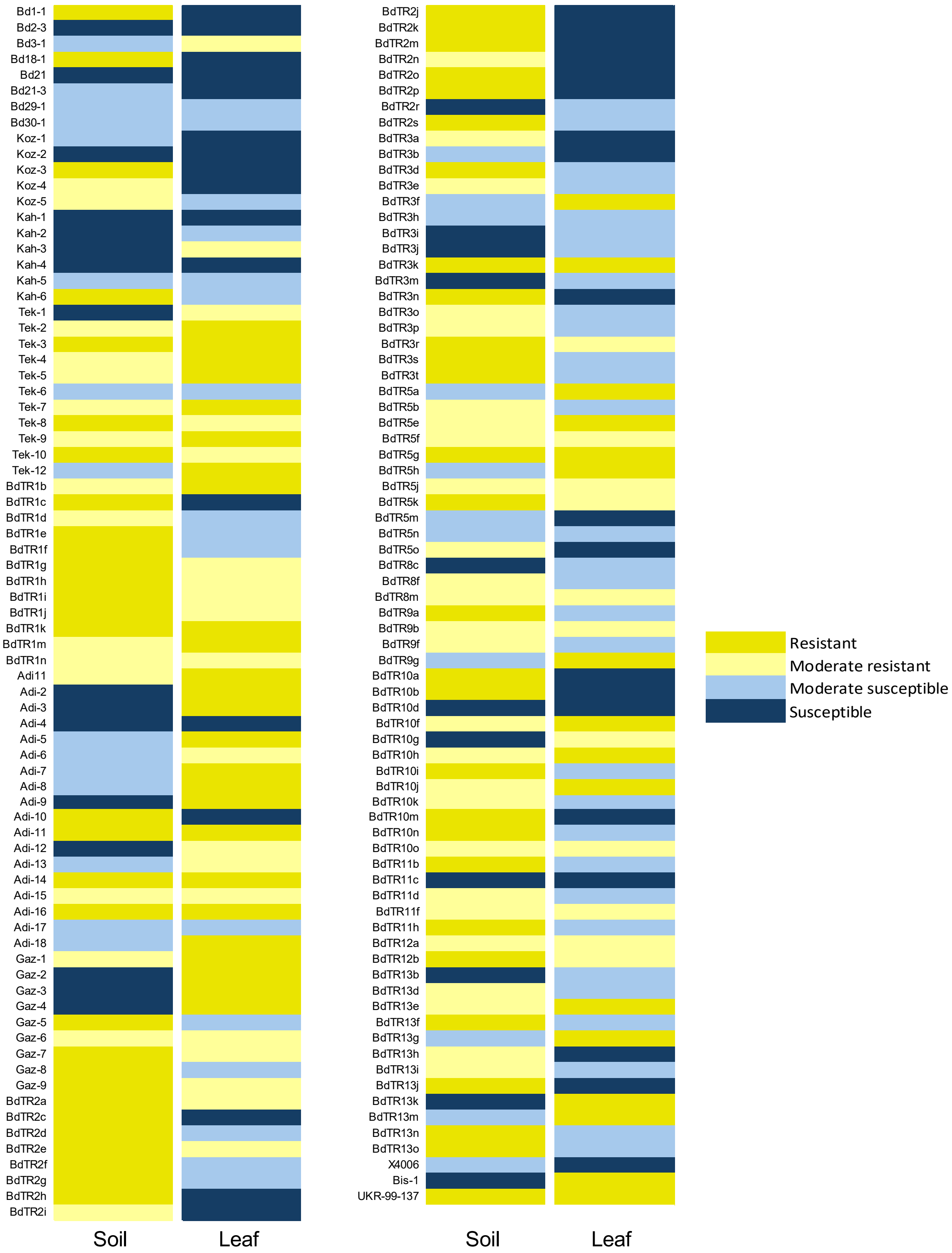

Supplementary Figure 2. Summary of disease resistance profiles of *Brachypodium distachyon* accessions to leaf and soil inoculation with *Rhizoctonia solani* AG-4 HG-I+II. Disease resistance levels in leaf and soil for 153 accessions to AG-4 HG-I+II are presented as a heat map (yellow, resistant (<15%); light yellow, moderately resistant (15–25%); light blue, moderately susceptible (25–50%); dark blue, susceptible (>50%)). Disease scores were evaluated at 3 and 7 days post-inoculations for leaf and soil assays, respectively.

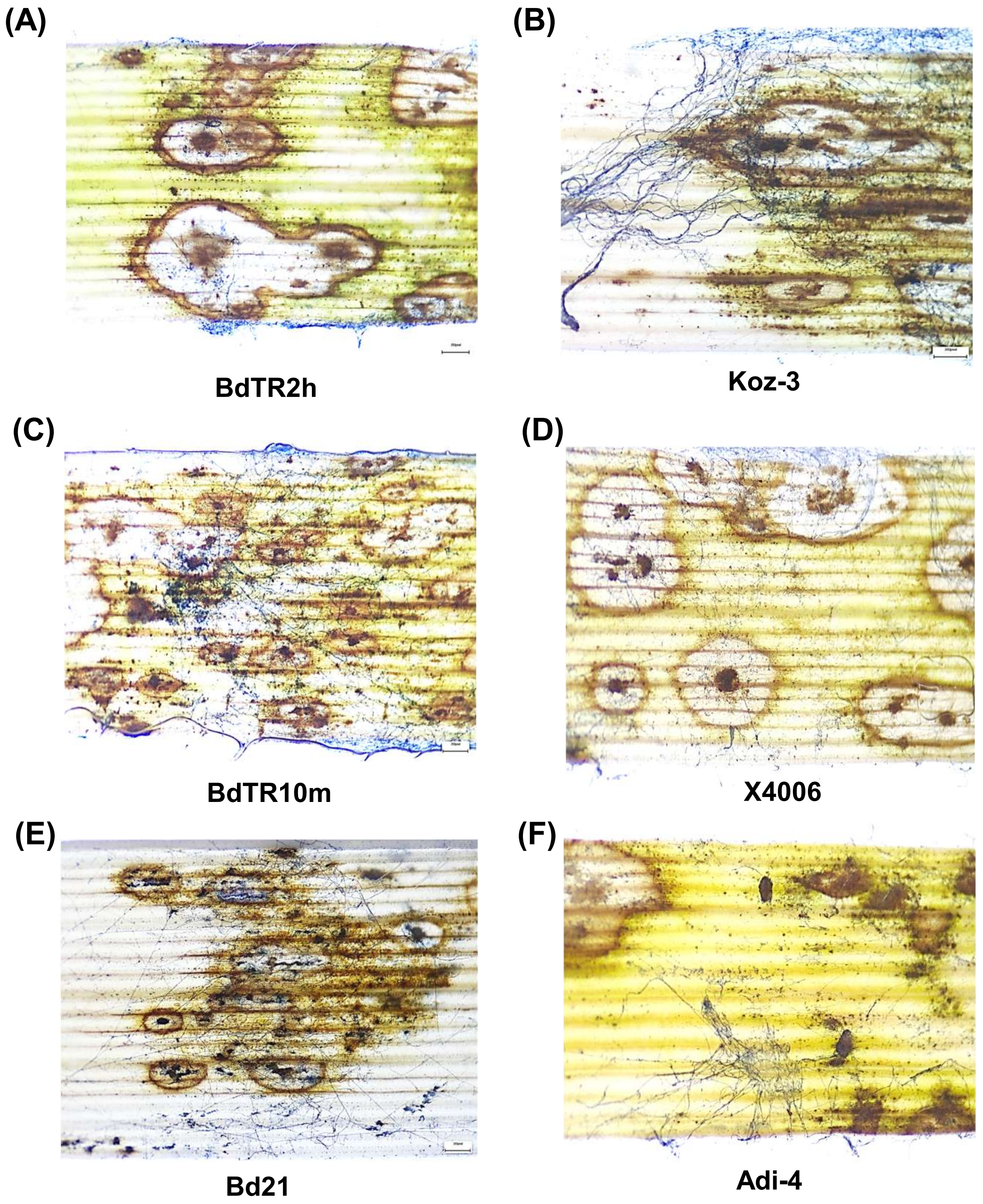

Supplementary Figure 3. Trypan blue staining of inoculated leaves from selected susceptible *Brachypodium distachyon* accessions following *Rhizoctonia solani* AG-4 HG-I+II infection. **(A–F)** Fungal hyphae and lesions stained with trypan blue in leaves of BdTR2h (A), Koz-3 (B), BdTR10m (C), X4006 (D), Bd21 (E), and Adi-4 (F) at 3 days post-inoculation with AG-4 HG-I+II. Brown discoloration was observed surrounding the necrotic lesions. Bar, 1 mm.

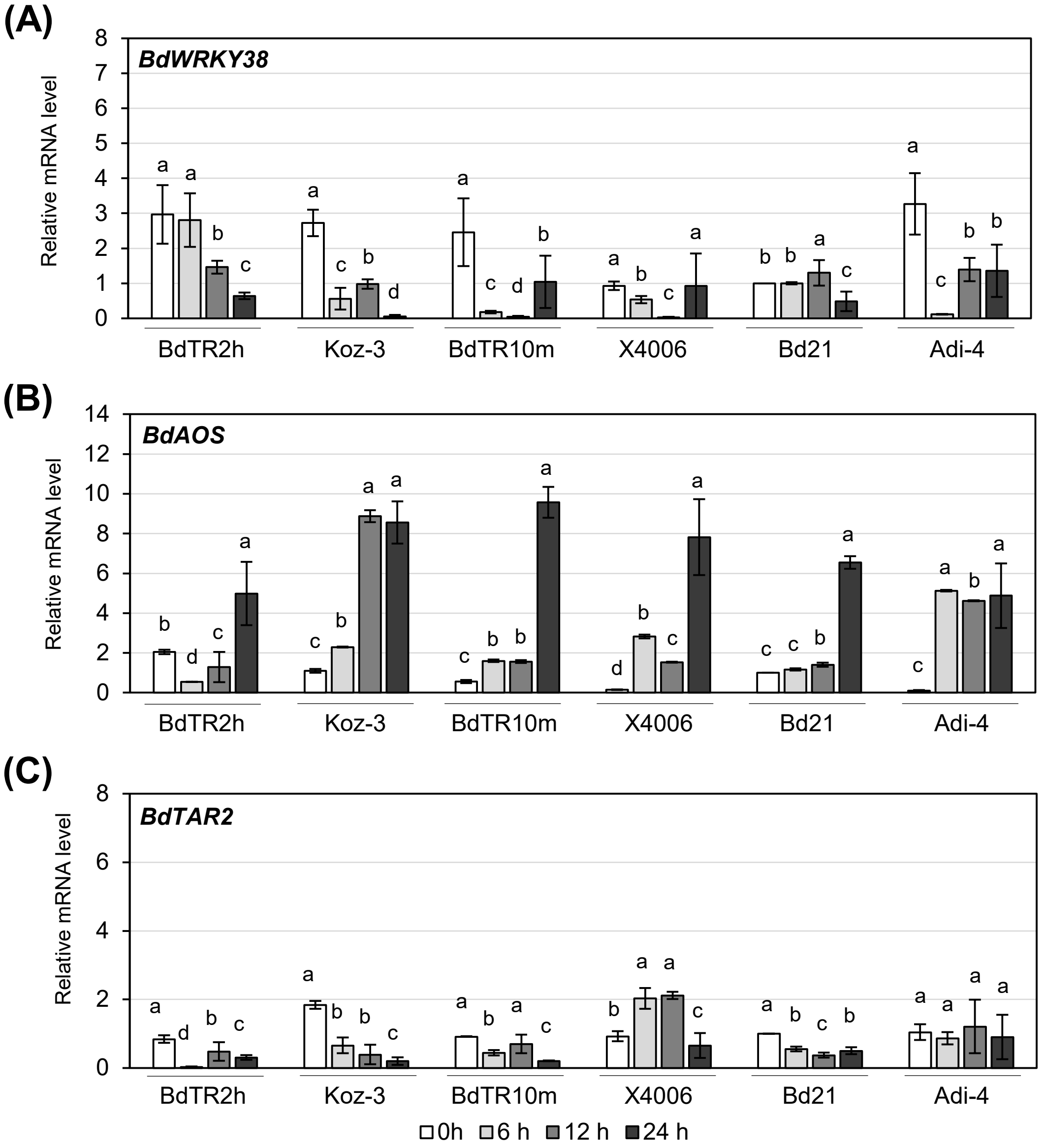

Supplementary Figure 4. Expression profiles of phytohormone-related genes in leaves of five highly susceptible *Brachypodium distachyon* accessions during *Rhizoctonia solani* AG-4 HG-I+II infection. **(A–C)** Relative mRNA levels of *BdWRKY38* (A), *BdAOS* (B), and *BdTAR2* (C), in leaves of *B. distachyon* accessions at 6, 12, 24 and 48 hours post-inoculation with AG-4 HG-I+II. Bd21 was used as a representative susceptible accession. Data are presented as mean values ± SD (n = 3). Different letters indicate statistically significant differences among time points within each accession (one-way ANOVA followed by Tukey's HSD post hoc test, $P < 0.05$).

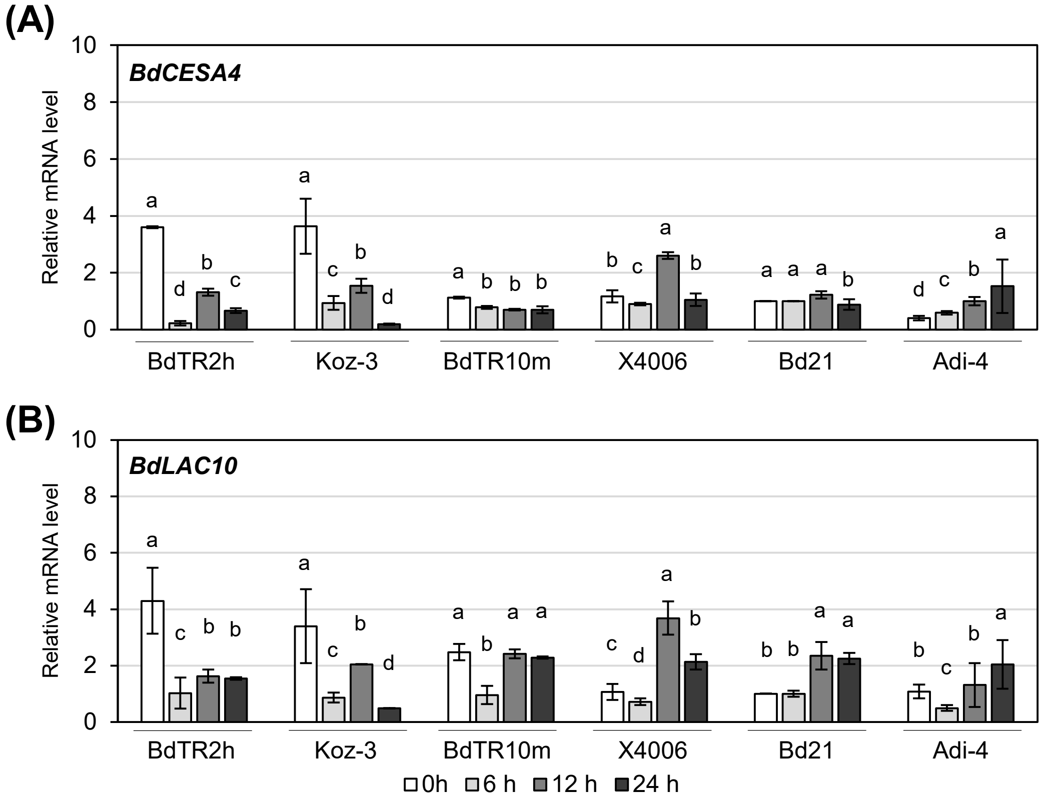

Supplementary Figure 5. Expression profiles of cell wall biosynthesis-related genes in leaves of five highly susceptible *Brachypodium distachyon* accessions during *Rhizoctonia solani* AG-4 HG-I+II infection. **(A,B)** Relative mRNA levels of *BdCESA4* (A) and *BdLAC10* (B), in leaves of *B. distachyon* accessions at 6, 12, 24 and 48 hours post-inoculation with AG-4 HG-I+II. Bd21 was used as a representative susceptible accession. Data are presented as mean values ± SD (n = 3). Different letters indicate statistically significant differences among time points within each accession (one-way ANOVA followed by Tukey's HSD post hoc test, $P < 0.05$).
